# Cutaneous pseudolymphoma: A clinicopathological study and immunohistochemical patterns

**DOI:** 10.22088/cjim.12.3.283

**Published:** 2021-04

**Authors:** Fatemeh Sari Aslani, Mozhdeh Sepaskhah, Akbar safaei, Sedigheh Jashounia Hormozi

**Affiliations:** 1 Molecular Dermatology Research Center, Shiraz University of Medical Sciences, Shiraz, Iran; 2 Department of pathology, Shiraz University of Medical Sciences, Shiraz, Iran

**Keywords:** Cutaneous, Pseudolymphoma, Immunohistochemistry

## Abstract

**Background:**

Cutaneous pseudolymphoma can histologically and clinically simulate various types of cutaneous lymphoma. We conducted the current study to evaluate the clinicopathological and immunohistochemical (IHC) aspects of cutaneous pseudolymphoma (PSL).

**Methods:**

30 cases of cutaneous PSL were selected from the archives of 2013-2017 in Shahid Faghihi Hospital pathology lab, Shiraz University of Medical Sciences. Available clinical data, histopathological features and IHC findings were statistically analyzed.

**Results:**

The female: male ratio was 2:1 and the median age was 47±14.9 years. The lesions were located on the head and neck 26 (86.7%), trunk 2 (6.7%) and extremities 2 (6.7%). 23 (76.7%) cases had nodular pattern, while 7 (23.3%) showed diffuse pattern. The grenz zone was seen in 24 (80%) cases. Sixteen (53.3%) cases showed top heavy infiltration, 11 (36.7%) showed the same distribution of infiltration at the superficial and deep dermis, often involving the subcutaneous fat and 3(10%) showed bottom heavy infiltration. In IHC, 11(36.6%) cases showed the B cell type, 10 (33.3%) T cell type and 9 (30%) mixed type (B and T cells).

**Conclusion:**

None of the cases was suspicious for cutaneous lymphoma, applying IHC staining. Gender distribution, and the site of cutaneous lesions were similar to previous studies. The most common histological subtype was nodular, while the B-cell cutaneous pseudolymphoma was slightly more common compared to the T-cell type.

Cutaneous pseudolymphoma, consisting of diverse groups of benign reactive T-cell or B-cell lymphoproliferative processes mimics cutaneous lymphoma. (1) Chronic persistent antigenic stimulation results in the proliferation of T cells and B cells. Some antigenic stimuli result in the proliferation of only B or T cells but some of them stimulate both B and T-cells. This leads to the formation of cutaneous lymphoid hyperplasia. (2) Cutaneous lymphoid infiltration can be seen in both benign and malignant conditions which causes a significant diagnostic challenge. Benign cutaneous lymphocytic infiltration includes classic inflammatory diseases such as psoriasis or lichen planus, and another group having none of the specific pictures of the classic inflammatory dermatoses. The latter group is termed PSL (3), which is a benign reactive T-cell or B-cell lymphoproliferative process, may mimic cutaneous lymphoma clinically and histologically. The differentiation between PSL and cutaneous lymphoma is usually difficult for both clinicians and pathologists.

As far as these two diseases have different therapeutic management and clinical course, it is necessary to properly differentiate between them. Misdiagnosis results in inappropriate chemotherapeutic treatment, and also causes psychological burden for the patients. (3) The diagnosis of PSL requires at least 4 mm diameter skin punch biopsy to be evaluated for histopathological findings. (4) In some cases of PSL, clinical findings and routine hematoxylin and eosin (H&E) examinations are inadequate to get the proper diagnosis; hence an ancillary study is required to differentiate it from cutaneous lymphoma. 

In this differentiation, IHC markers are helpful, but no single marker is sensitive or specific enough for the diagnosis of PSL. So for correct differentiation, a panel of IHC markers is required. (5, 6) In this study, a panel consisting of CD3, CD5, CD7, CD4, CD8, CD 20, CD30, BCL2 and Ki67 was used for this differentiation. This study included the clinical and histopathological findings and also IHC features of PSL, to strengthen the clinicopathological diagnosis of PSL.

## Methods

**Patient selection: **In this cross-sectional study, we searched the archives of the surgical pathology lab of the Shahid Faghihi Hospital of Shiraz University of Medical Sciences for cases of cutaneous PSL or cutaneous lymphoid hyperplasia, between 2013 and 2017. A total of 30 cases were selected. For confirmation of the diagnosis, a dermatopathologist reviewed the hematoxylin and eosin (H&E) slides. The histomorphological criteria used for diagnosis were based on Weedon’s skin pathology (7), including epidermal reactive changes (such as spongiosis, acanthosis), presence or absence of the grenz zone between the epidermis and dermis, pattern recognition on low power including infiltration of the lymphoid cells in the nodular (lymphoid follicle formation) or diffuse (dermal infiltration of benign looking lymphoid cells without formation of distinct nodules) patterns in the B cell PSL. T cell PSL is characterized by superficial band like, nodular or diffuse patterns admixed with dermal histiocytes, eosinophils and plasma cells infiltrating, mainly the upper dermis (top heavy distribution). (7) The histological features of each case as well as the clinical information including age, sex, site of lesion were recorded.

**Exclusion criteria: **Specimens with inadequate tissue for IHC study, the slides which do not fulfill the criteria of PSL (7) and the cases with inadequate clinical data were excluded 

from the study. 

**Immunohistochemical technique of staining: **Immunohistochemical examination for CD3, CD5, CD7, CD4, CD8, CD20, CD30, BCL2 and Ki67 were performed on tissue sections from formalin-fixed (neutral buffered 10% formalin), paraffin embedded blocks. For deparaffinization, incubation in Fur (T=60°C, 3 times, each 10 min) was used, followed by gradual rehydration in ethanol, and phosphate buffer saline wash for 5 min. The antigen retrieval step was done in TRIS buffer (PH=9) for 15 min in 100°C. All the slides were incubated in H_2_O_2_ 3% for 5 min, followed by the use of DAKOPEN. 

Then, the slides were incubated for 50 min with optimally ready-to-use antibodies including CD3 (clone EP41), CD5 (clone 4C7) CD7 (clone EP132), CD4 (clone EP204), CD8 (clone SP16), CD20 (clone L26), CD30 (clone Ber-H2), BCL2 (clone 100/D2), and Ki67 (clone SP6). The following steps included phosphate buffer saline wash (2 times, 5 min each time), using a primary antibodies amplifier for 15 min and slide wash with phosphate buffer saline (2 times, 5 min each time), adding polymer HRP for 30 min, and again, phosphate buffer saline wash (2 times, 5 min each time). Thereafter, DAB (3, 3 Diaminobenzidine) chromogen was added for 5 min. CD3,5 and BCL-2 were obtained from Biocare and the others from Master Diagnostica. Cellular localization for ki67 was nuclear, cytoplasmic while cell membrane and cytoplasmic for others.

At the final step, after counterstaining with hematoxylin, and rinsing in running water, the slides were dehydrated in graded ethanol solutions. Xylene cleared the slides and then they were mounted. The IHC criteria used for the diagnosis of B cell PSL were the expression of small CD20 positive B lymphocytes in the reactive lymphoid follicles, BCL2 expression in mantle zone and interfollicular regions, similar expression of CD3, CD5 and CD7 in the inter follicular regions, high expression of Ki67 in the germinal center and the low expression of it in other regions (8, 9).

The IHC criteria for T-cell PSL included the absence of an aberrant immunophenotype. The T-cells show immunopositivity for CD3, CD5, CD4, and CD8. CD4 positive intraepidermal lymphocytes showed positive staining for CD3, CD5, and CD7. CD4/CD8 ratio was 1/1 to 3/1. The CD8/CD3 ratio was less than 50% (8, 9).

**Statistical analysis:** We analyzed data using the SPSS software Version 22. Descriptive statistics was calculated and then the results were evaluated by Fisher's Exact Test.

## Results

Table 1 contains the clinical data of the cases.

**Table 1:** Gender, site of the lesions, and age range of the cases of cutaneous PSL

**Clinical parameters**		**No**	**%**
No. of patients		30	100
Gender	Female	20	66.7
	Male	10	33.3
Site of lesion	Head and neck	26	86.6
	Trunk	2	6.7
	Extremities	2	6.7
Age range (year)		19-87	
Age Median (year)		47±14.9	

Clinical impression of PSL was noted in all the cases as the only diagnosis or one of the differential diagnoses. The histopathological features included the infiltration of small and large lymphocytes, and histiocytes admixed with some eosinophils and plasma cells. Some cases showed a germinal center containing small cleaved and non-cleaved lymphoid cells surrounded by a well-formed mantle zone. The interfollicular space showed the infiltration of small lymphocytes, some eosinophils and plasma cells. We categorized the histological data into 3 groups: nodular with germinal center, nodular without germinal center and diffuse pattern. The histological subtypes and histomorphological findings are presented in table 2.

**Table 2:** Histologic subtypes and histomorphological findings of cutaneous PSL

Histological findings		No	%
Histological subtype	Nodular with germinal center	11	36.7
	Nodular without germinal center	12	40
	Diffuse	7	23.3
Histomorphological findings	Grenz zone	24	80
	Epidermal changes	14	46.7
	Exocytosis of lymphocytes	6	20
	Top heavy	16	53.3
	Top and bottom heavy	11	36.7
	Bottom heavy	3	10

We categorized IHC findings into 4 groups: B-cell PSL with a germinal center, B-cell without a germinal center, T cell, and mixed type (B and T cells) presented in table 3. 

**Table 3:** Subtypes of PSL based on IHC features

**Classification**	**No**	**%**
B cell type with germinal center	10	33.3
B cell type without germinal center	1	3.4
T cell type	10	33.3
Mixed type (B and T)	9	30

**Figure 1:**
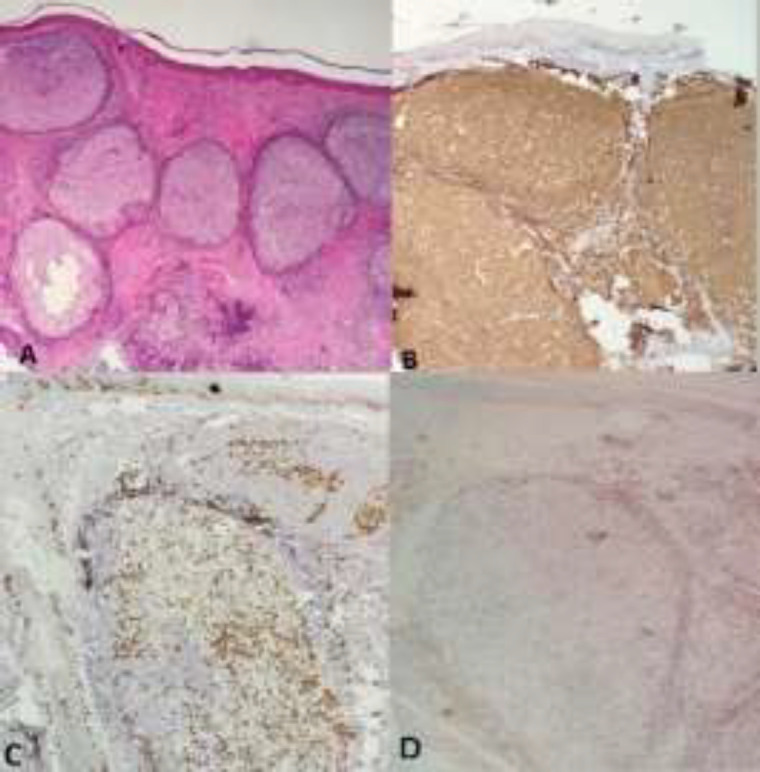
(A) B cell typeshowed lymphoid follicles with germinal center (H&Ex100), (B) CD20 was positive in the germinal center and scattered in the perivascular and periadnexal regions. (×200), (C) Ki67 was high in the germinal center and low in other parts (x 200), (D) CD3 was positive in the interfollicular, perivascular and periadnexal areas (x200)

In B-cell PSL with a germinal center, CD20 was positive in the germinal center and scattered in the perivascular and periadnexalregions. CD3, CD5, and CD7 were positive in the interfollicular, perivascular and periadnexal areas. BCL2 was positive in the mantel zone, marginal zone and interfollicular areas and was negative in the germinal center. Ki67 was high in the germinal center and low in other parts (Figure 1). 

**Figure 2:**
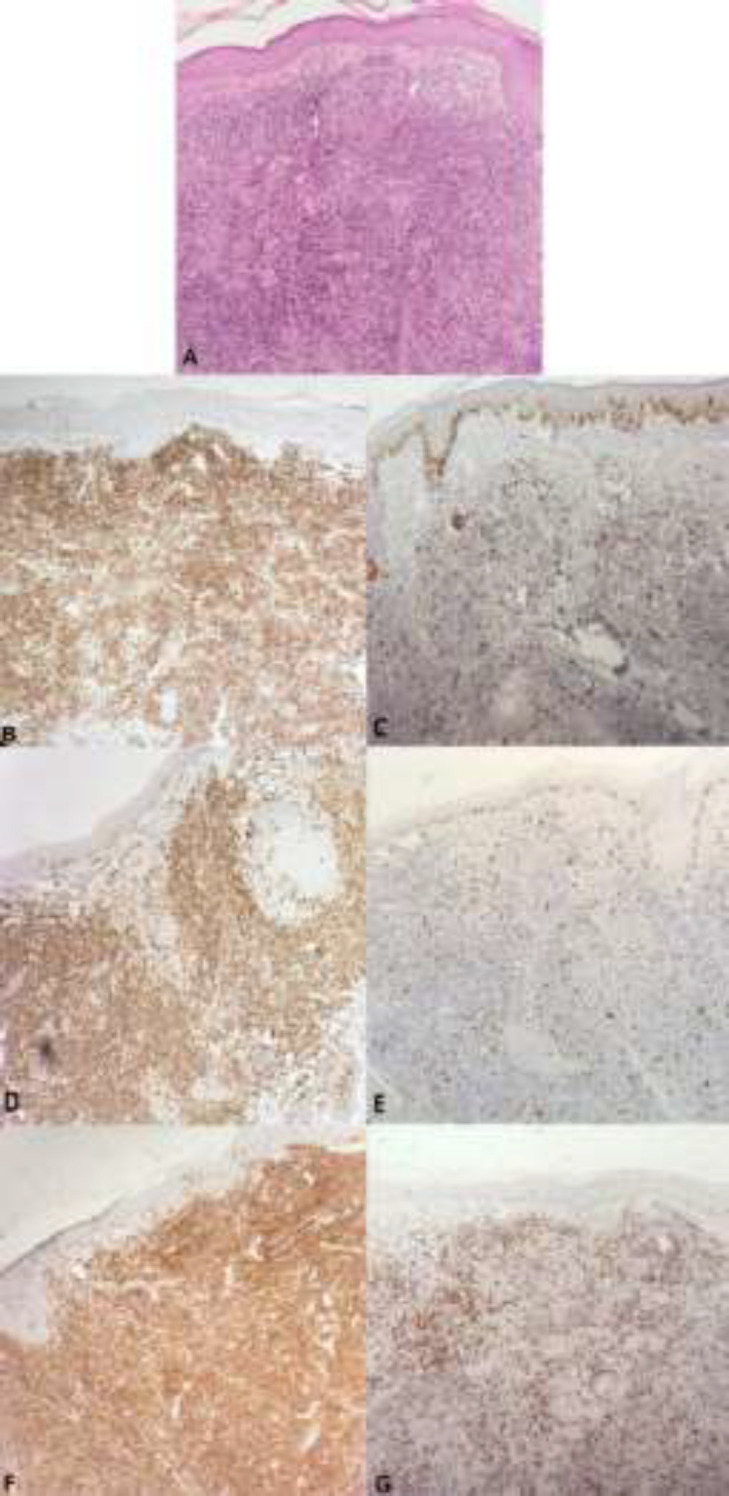
(A)T cell type showed diffuse infiltration of chiefly lymphocytes mainly in the upper dermis with mild exocytosis into the epidermis (H&Ex100), (B) CD3 was positive in the perivascular and periadnexal regions (x100), (C) CD20 was scatteredly positive (x100), (D) CD7 was scatteredly positive in the perivascular and periadnexal areas (x100), (E) Ki67 is low (x100), (F and G) CD4 and CD8 were positive in T cells and CD4 positive cells are more than CD8 positive cells (x100)

**Figure 3:**
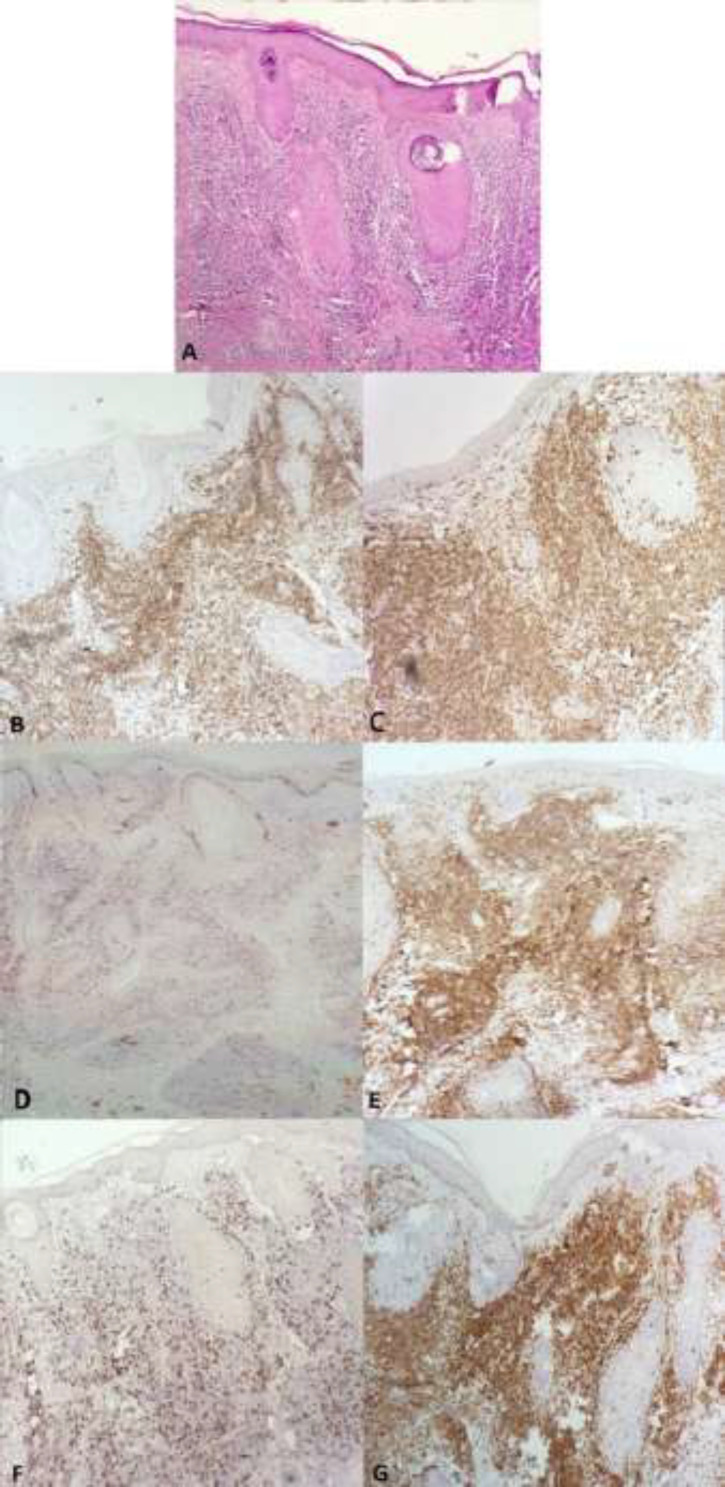
(A) Mixed type showed dense perivascular, periadnexal and interstitial infiltration of mainly lymphocytes (H&Ex100), (B and C) mixture of CD3 and CD20 positive cells (x100), (D) Ki67 is low (x100), (E and F) CD4 and CD8 are positive in T cells and CD4 positive cells are more than CD8 positive cells (x100), (G) BCL2 positive in B and T cells (x100)

CD30 was scattered positively in immunoblasts. In B-cell PSL without a germinal center, CD20 was positive in B lymphoid aggregates, perivascular and periadnexal areas. CD3, CD5, and CD7 were positive scattered data. BCL2 was expressed in the lymphoid aggregate and interfollicular regions. CD30 was scatteredly positive in immunoblasts and Ki67 was low. In T-cell PSL, CD20 was scatteredly positive. CD3, CD5, and CD7 were positive in the perivascular and periadnexal regions and BCL2 also was positive in the perivascular and periadnexal areas. CD30 was positive in immunoblasts. The CD4/CD8 ratio was 2/1 in 5 cases, 4/1 in 3 cases and 3/1 in one case. It was found that Ki67 is low (fig. 2). In the mixed type (B and T cells) lymphocytes were positive for B and T-cell markers. CD30 was scatteredly positive in immunoblasts.Ki67 was low.CD3, CD5 and CD7 were scatteredly positive in the perivascular and periadnexal areas (fig. 3).

## Discussion

Cutaneous PSL has a wide range of clinicopathological presentations which mimic the cutaneous lymphoma. The clinical presentation is principal to convey and establish a differential diagnosis of neoplastic and reactive lymphoid infiltrates (10). Pseudolymphoma and lymphoma have different clinical courses and treatments, hence, it is necessary to differentiate between them (3). Pseudolymphoma is more common in females (11). In our study, there were 20 (66.7%) females and 10 (33.3%) males with F/M ratio of 2/1. In Bergman et al.'s study, there were 9 (37.5%) females and 15 (62.5%) males (M/F ratio: 1.6). (10) In a review of the reported cases of acral pseudolymphomatous angiokeratoma of children (APACHE), Tokuda et al. found 77% female patients, having 52% of lesions in the acral area and 70% of the affected individuals consisted of children and adolescents (12). Cutaneous PSL can be seen in a wide age range (7) but is more common in people less than 40 years of age (13). Our patients were 19 to 87-year-old (median age: 47 years). In Bergman et al.'s study, the age range of the patients was between 6 to 93 years (median: 54 years) (14). In Cerroni L et al.'s case series of 4 patients that developed B-cell PSL after vaccination were 19 to 60 years old (median: 34.5 years) and all patients were females (15). Pseudolymphoma most commonly present on the face (cheeks, nose, and earlobe), chest, and upper extremities (7). In our study, the most common site was the face (73.3%), followed by the scalp (13.3%), trunk (6.7%) and extremities (6.7%). In Bergman et al.'s study with 24 cases of PSL, the lesions were located on the head and neck (50%), trunk (41.7%), and extremities (8.3%) (14). 

Several algorithms were presented in the previous articles to provide a practical approach for the diagnosis of cutaneous lymphoproliferative disorders (10, 17). The algorithmic approach to cutaneous lymphoid infiltrates needed to be applied together with essential clinical contributions, histopathological pattern on low power, cytomorphology of cells in terms of size and nuclear characteristic, and interpretation of the appropriate IHC panels (18).

 The approach to reactive cutaneous lymphoid infiltrates should include the recognition of the predominant pattern to be epidermotropic, dermal-based, or mostly subcutaneous. Such pattern recognition can be helpful in the consideration of a list of differential diagnoses which may be confused with both T- and B-cell lymphomas (10). In our study we also applied a systematic approach to confirm the diagnosis according to Weedon’s text book considering morphologic features (pattern recognition on low power), cytomorphology of cells, and immunophenotypic evaluation. 

Histologically, PSL may have a nodular or diffuse pattern. In this study, we categorized histological data into 3 groups: 1) nodular with germinal center, 2) nodular without germinal center and 3) diffuse pattern. A total of 12 (40%) cases were nodular without a germinal center pattern, 11 (36.7%) cases were nodular with a germinal center pattern and 7 (23.3%) cases had a diffuse pattern. In Bergman et al.'s study, 16 (66.7%) cases were nodular with a germinal center pattern, 5 (20.8%) cases were nodular without a germinal center pattern and 3 (12.5%) cases had a diffuse pattern (10). In Eiichi Arai et al.'s study, 18 cases of PSL showed a diffuse pattern and 11 cases had a patchy distribution (16).

In PSL, infiltration is mainly present in the upper dermis (top heavy distribution) but in some cases, lymphoid infiltration may be seen in the deeper dermis and also in subcutaneous fat. (7) In our study, 16 (53.3%) cases showed top heavy infiltration, 11 (36.7%) cases showed the same distribution of infiltration at the top and the bottom, often extending to subcutaneous fat and 3 (10%) cases showed bottom heavy infiltration. In Bergman et al.'s study, 12 (50%) cases showed top heavy infiltration, 11 (46%) cases showed the same distribution of infiltration at the top and bottom and 1 (4%) case showed bottom heavy infiltration (12). In Boer et al.'s study, 17 (56.6%) cases had top heavy infiltration and 5 (16.6%) cases showed bottom heavy infiltration. (19) Cutaneous PSL can be classified into B-cell, T-cell and mixed (B and T-cell) types. (20) We recognized the B-cell type in 11 (36.7%) cases of our cases, with a germinal center in 10 (33.3%) cases, and without a germinal center in 1 (3.4%) case. The T cell type was seen in 10 (33.3%) cases whereas the mixed type (B and T-cell) was seen in 9 (30%) cases. In Bergman et al.'s study, a B-cell type with a germinal center was seen in 16 66.7%) cases (, T-cell type in 5 (20.8%) cases and mixed type (B and T-cell) was seen in 3 (12.5%) cases. Eiichi Arai et al, reevaluated 55 cases of non-epidermotropic lymphoproliferative disorder for several IHC markers and the gene rearrangement of the immunoglobulin heavy chain. These cases had been labeled as cutaneous PSL in the initial examination. The new diagnoses included, cutaneous marginal zone lymphoma (4 cases), pseudolymphomatous folliculitis (PLF) (19 cases), diffuse large B-cell lymphoma (1 case), solitary non-epidermotropic pseudo-T-cell lymphoma (2 cases), and cutaneous lymphoid hyperplasia (CLH) (29 cases). Cutaneous marginal zone lymphoma (7.3% of the cases) was differentiated from CLH by proliferation of centrocyte-like cells (patchy or diffuse), plasma cells encircling the lymphocytic infiltration, light chain monotypic restriction , and gene rearrangement of the immunoglobulin heavy chain. The authors defined the presence of irregular morphology of hair follicles, infiltrated and surrounded by CD1a-and S-100 protein-positive T-cell-activated dendritic cells, as diagnostic criteria of pseudolymphomatous folliculitis (34.5% of cases) (16). The IHC panels used in previous studies were slightly different. In Bergman et al.'s study, the IHC markers included CD20, CD30, CD21, BCL6, BCL2, CD10, CD3, CD68, CD1a, Ki67, S100 protein, and kappa and lambda light chains. (7) In Tadashi Terada's study, the IHC panel included CD20, CD23, CD10, CD79a, BCL2, BCL6, CD3, CD4, CD5, CD8, CD43, CD45RO, CD56, CD57, CD38, CD138, Ki67, and kappa and lambda light chains.(18) In Boer A et al.'s study, 30 histological specimen of pseudolymphomatous cutaneous infiltrates were selected. The IHC panel included CD1a, CD3, CD4, CD8, CD20, CD30, CD10, BCL6, CD68, and CD138. Rearrangement study findings included, B-cell pseudoclonality (7 cases), T-cell pseudoclonality (4 cases) and B-cell clonality (4 cases). Both B-cell pseudoclonality and B-cell clonality were present in moderately dense infiltrates and was shown only in a few B lymphocytes. The predominance of T-cell pseudoclonality was also in moderately dense infiltrates. Moderately dense pseudolymphomatous infiltrates may contain B-cell pseudoclones (23%) and T-cell pseudoclones (13%). Pseudolymphomatous infiltrates (especially B lymphocyte -poor ones) may demonstrate clonal B-cells (13%). Therefore, the authors discourage the interpretation of rearrangement studies independent of morphological patterns. They suggest performing multiple confirmatory immunophenotyping tests to prevent misinterpretation (19).

Cutaneous PSL can progress into a lymphoma. (7) Nihal et al. utilized the IHC and clonality study in 44 PSL patients. Thirty-eight typical mixed B-cell/T-cell type and 6 T-cell-rich type (more than 90% T cells) were studied. Thirty-eight cases presented solitary or localized lesions (4 cases of T-pseudolymphoma), and 6 showed regional/generalized lesions (2 cases of T- PSL). Overall, 27 specimen (61%) showed clonal PSL: 12 IgH+. After 24 months of follow-up, two (4%) cases, one monoclonal and another polyclonal proceeded to the cutaneous B-cell lymphoma. The authors concluded that proliferation of clonal populations is usual in CLH. They considered the sensitivity of PCR and patient selection as a possible cause of high prevalence of dominant clonality in their study (1). Cutaneous lymphocytic infiltrates are common in numerous neoplastic and inflammatory dermatoses and encompass some of the most challenging entities in dermatopathology. Immunophenotypic and molecular techniques have improved their proper classification. However, as a result of the specificity and sensitivity limitations of ancillary techniques, the diagnosis of cutaneous lymphomas and simulants continues to rest on the clinicopathological correlation with IHC and clonality studies serve as adjunctive tools in some cases. The challenging cases, may ultimately benefit from referral to dedicated cutaneous lymphoma centers for expert evaluation (3).

In our study, the most common histological subtype was nodular. None of our cases were suspicious for the neoplastic process on IHC study. The most common histopathological features were the presence of grenz zone in B -cell PSL. The B-cell type was slightly more common than the T- cell type. The results of this study can be helpful in providing knowledge on the clinical, histopathological and IHC features of PSL which leading to a more exact diagnosis of PSL by dermatologists and dermatopathologists.
